# Victory Over Pediatric Chronic Calcaneal Osteomyelitis With Maintenance of Vancomycin-Permeated Bone Spacer: A Case Avoiding Second-Stage Surgery

**DOI:** 10.7759/cureus.86888

**Published:** 2025-06-27

**Authors:** Wynnie Hui Yin Voon, Muhamad Nurasnol Khamis

**Affiliations:** 1 Orthopedic, Hospital Tawau, Tawau, MYS; 2 Orthopedics, Hospital Tawau, Tawau, MYS

**Keywords:** calcaneal osteomyelitis, chronic recurrent osteomyelitis, osteomyelitis diagnosis, osteomyelitis of the calcaneus, paediatric calcaneum osteomyelitis, pediatric chronic osteomyelitis, pediatric osteomyelitis, polymethylmethacrylate (pmma), vancomycin-impregnated pmma cement, vancomycin powder

## Abstract

Chronic calcaneal osteomyelitis in pediatric patients is challenging, with high recurrence rates and limited treatment options. This case report describes an eight-year-old girl with persistent symptoms following a fall. Despite initial antibiotic therapy and debridement, imaging revealed cortical disruption and sequestrum, leading to a diagnosis of chronic osteomyelitis. Surgical debridement and sequestrectomy were followed by vancomycin-impregnated polymethylmethacrylate (PMMA) cement for infection control and mechanical support. A six-week course of oral antibiotics was administered. At three-year follow-up, the patient remained symptom-free without the need for second-stage surgery to remove the PMMA cement. This case illustrates the success of single-stage, antibiotic-loaded PMMA cement in treating pediatric chronic calcaneal osteomyelitis, yielding long-lasting functional and infection-free outcomes. The use of PMMA cement offers key advantages, such as excellent biocompatibility, ease of handling, straightforward processability, and cost-effectiveness.

## Introduction

Chronic calcaneal osteomyelitis presents significant treatment challenges due to its high recurrence rates and the complexity of managing bone infections, especially in pediatric patients. Successful treatment requires a multidisciplinary approach, involving orthopedic surgeons, infectious disease specialists, and occasionally plastic surgeons, to address both the infection and the preservation of bone integrity. While antibiotic-impregnated cement has proven effective in treating osteomyelitis [1], its use in pediatric cases is still underexplored, warranting further investigation into its long-term safety and efficacy.

Effective management of calcaneal osteomyelitis requires a comprehensive strategy that includes complete debridement of infected and necrotic tissue, dead space management, and the preservation of weight-bearing function to maintain gait and mobility. Additionally, ensuring adequate soft tissue coverage is critical to creating a vascularized environment that supports healing. A key challenge in managing this condition is the need for a local antibiotic delivery system capable of effectively targeting residual infection while minimizing systemic toxicity.

Antibiotic bone cement spacers come in two main types: non-absorbable and absorbable. Non-absorbable spacers, such as those made from polymethylmethacrylate (PMMA), are favored for their mechanical strength and have been widely used in orthopedic practice to treat osteomyelitis. PMMA spacers offer strong structural support to the infected site, but they require second-stage surgery for removal after four to six weeks to prevent biofilm formation, chronic inflammation, and reinfection [2]. This can pose challenges for patients who are medically unstable or encounter logistical difficulties in attending follow-up care.

On the other hand, absorbable spacers, typically made of calcium sulfate or other biodegradable materials, offer the advantage of eliminating the need for a second-stage surgery. However, these spacers often have lower mechanical strength compared to PMMA and may not offer the same level of support [3]. Furthermore, absorbable spacers are often more expensive, which can limit their accessibility, particularly in resource-limited settings [3].

In this case, we describe a pediatric patient with chronic calcaneal osteomyelitis who was successfully treated with vancomycin-impregnated PMMA cement. Despite retaining the antibiotic-loaded PMMA cement spacer for three years, the patient showed excellent functional recovery, with no signs of infection. This case contributes to the growing body of evidence suggesting that PMMA cement spacers can be retained long-term without significant complications, provided the infection is adequately controlled [1].

## Case presentation

An eight-year-old previously healthy girl presented to Hospital Tawau, Sabah, Malaysia, in 2022 with a two-week history of swelling and persistent seropurulent discharge from the right heel following a traumatic fall. She had initially been managed by a general practitioner with a one-week course of oral Cloxacillin, but her symptoms did not improve, prompting referral for further evaluation.

Initially, the injury was associated with mild discomfort, which progressively worsened. Over time, she developed increasing swelling and a punctum on the lateral aspect of the right heel, accompanied by continuous serous discharge. Upon presentation, the patient was afebrile with stable vital signs and appeared systemically well.

Laboratory investigations revealed a white blood cell count of 10 × 10³/μL, a C-reactive protein (CRP) level of 12.5 mg/L, and an erythrocyte sedimentation rate (ESR) of 89 mm/hr. Surgical wound debridement was performed to manage the local infection and remove necrotic tissue. Intraoperative samples were sent for microbiological analysis, including bacterial cultures and acid-fast bacilli (AFB) testing, both of which returned negative results.

Despite initial surgical intervention and continued oral Cloxacillin, the wound failed to heal, and symptoms persisted, raising suspicion for a deeper underlying infection. After five months of ongoing discharge and non-healing, a second surgical procedure was undertaken, involving wound debridement, calcaneal corticotomy, and insertion of Collatamp, a gentamicin-impregnated collagen sponge. Intraoperative cultures again yielded no pathogenic organisms. Due to compliance issues with the four-times-daily dosing schedule of Cloxacillin, the antibiotic regimen was changed to oral Augmentin syrup (amoxicillin-clavulanate) 500 mg twice daily.

**Figure 1:**
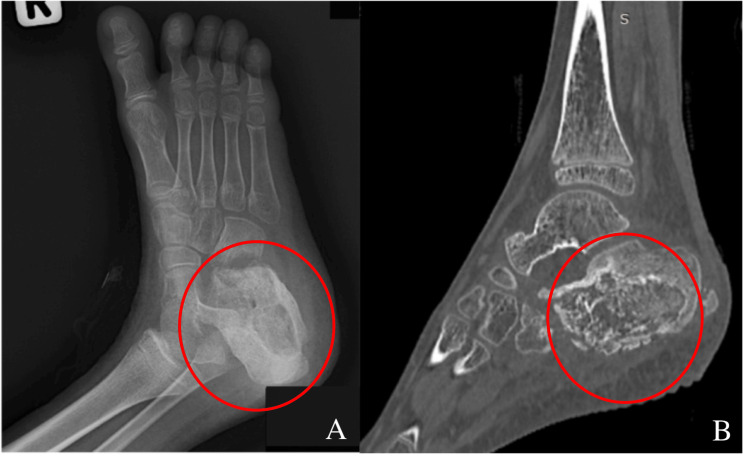
Preoperative radiograph of patient's right foot in oblique view (A) and CT image in sagittal plane (B) The lytic lesions and sequestrum, highlighted in red, show the bone destruction caused by osteomyelitis.

Despite continued antibiotic therapy, the wound remained non-healing with persistent seropurulent discharge 11 months after the initial presentation. Laboratory investigations revealed a CRP level of 80 mg/L and an ESR of 97 mm/hr. Further evaluation with imaging was conducted, including preoperative computed tomography (CT) of the right heel (Figure 1), which revealed significant bony involvement. The findings included cortical disruption of the anterior and plantar aspects of the calcaneus, the presence of an intraosseous sequestrum surrounded by an involucrum, and a cloaca at the posterior calcaneus. Additionally, a sinus tract extending to the heel surface was noted, along with cortical irregularity and erosion of the adjacent cuboid bone, associated with adjacent fat streakiness. These imaging findings were consistent with chronic osteomyelitis of the calcaneus.

**Figure 2:**
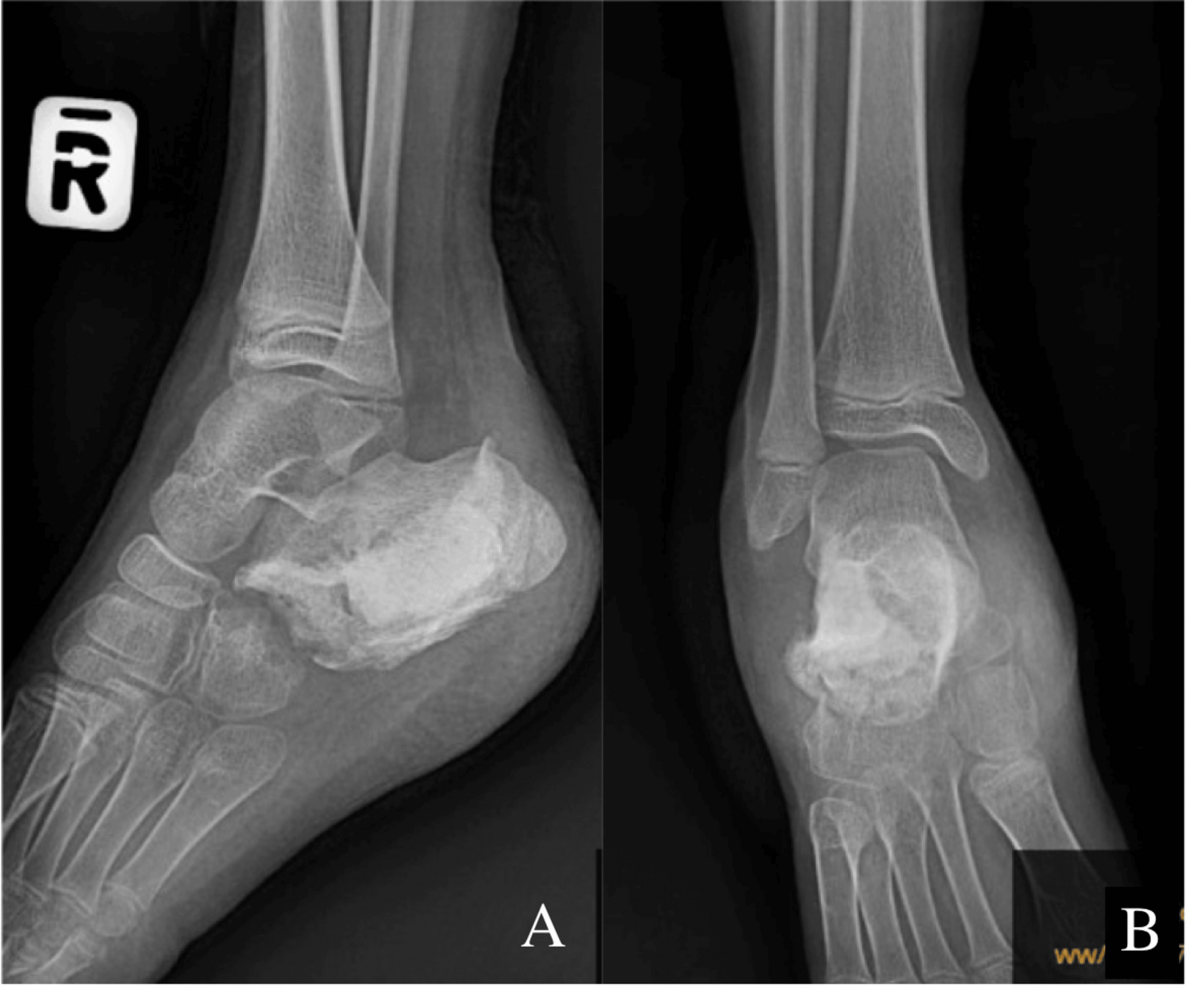
Postoperative radiographs with antibiotic bone cement filling the cavity showing in lateral (A) and anteroposterior (B) view

Given the severity of the infection and the failure of conservative measures, the patient underwent another surgical procedure, which included wound debridement and sequestrectomy. To address the resultant dead space and provide local infection control, vancomycin-impregnated PMMA cement was used (Figure 2). Specifically, 2 grams of vancomycin were mixed with 40 grams of PMMA cement, as per recommended concentrations for orthopedic infections. This approach provided high local antibiotic concentrations while minimizing systemic toxicity.

Postoperatively, the patient was prescribed intravenous (IV) Vancomycin 10 mg/kg QID. However, she developed an allergic reaction characterized by periorbital edema and facial rashes. Consequently, IV therapy was switched to IV Clindamycin 15 mg/kg QID and IV Cloxacillin 25 mg/kg QID. Tissue samples obtained during debridement identified Streptococcus pyogenes, which was sensitive to Ampicillin, Clindamycin, Penicillin, and Erythromycin.

Following a two-week course of intravenous antibiotics, the patient was discharged on oral Cloxacillin 25 mg/kg QID and oral Clindamycin 40 mg/kg divided into three doses daily. She was closely monitored at the outpatient clinic, with regular follow-up to track her inflammatory markers.

Two months after the antibiotic cement spacer insertion, the oral antibiotics were discontinued as the wound healed significantly. Laboratory markers improved, with a CRP level of 5 mg/L and an ESR of 41 mm/hr. Due to socioeconomic constraints, a second-stage surgery to remove the PMMA cement and insert bone substitutes was not performed. Instead, the patient was closely monitored for potential complications.

**Figure 3:**
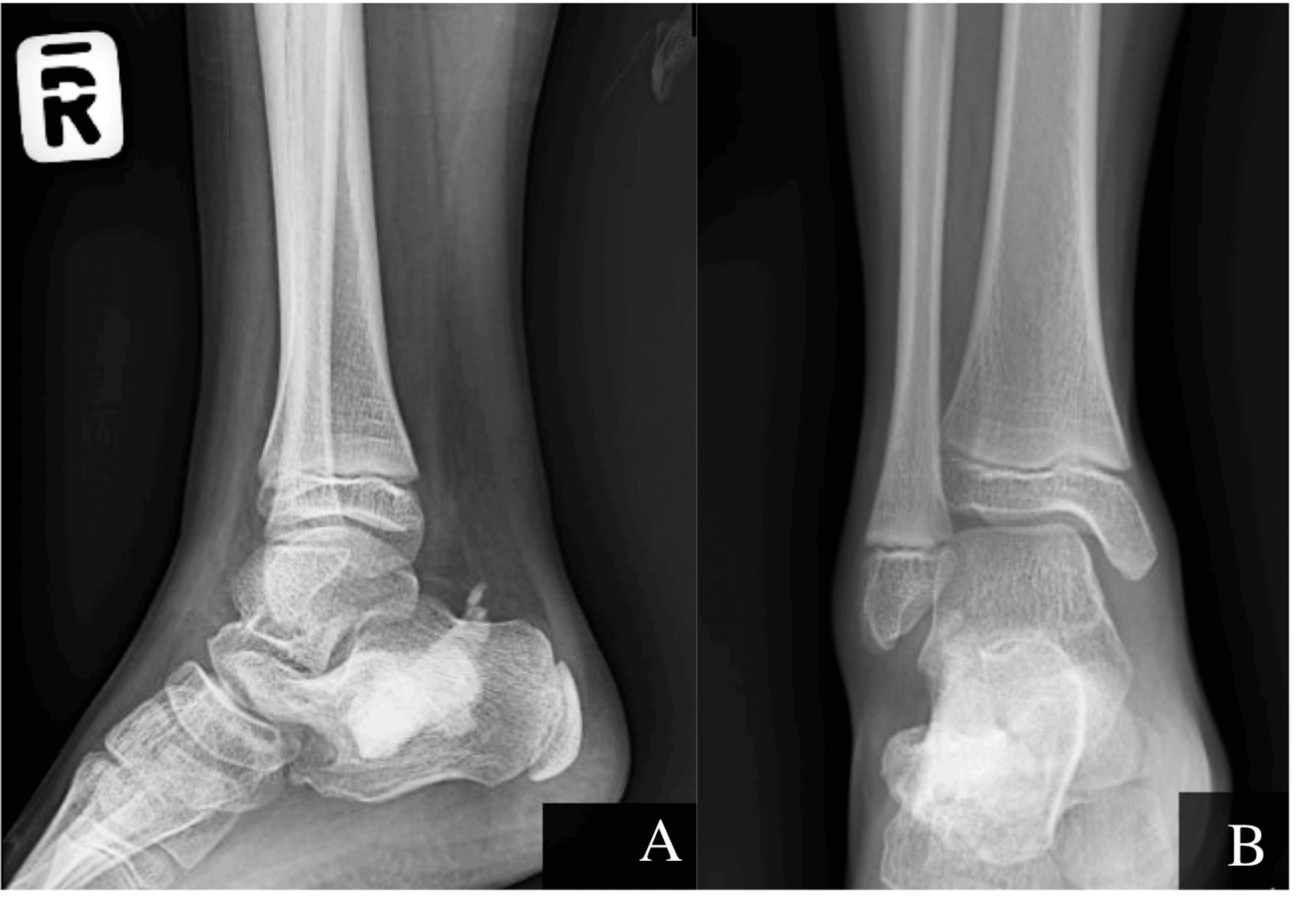
Postoperative radiographs in the lateral (A) and anteroposterior (B) views, showing the antibiotic bone cement spacer in place after 3 years

Three years post-surgery, the patient made a full recovery. She remained asymptomatic, pain-free, and was able to walk independently without assistance. She returned to school and resumed normal activities. Follow-up radiographs (Figure 3) demonstrated no signs of infection or complications related to the retained PMMA cement, with good healing of the calcaneus. Her most recent laboratory results, including a CRP level of 3 mg/L and an ESR of 5 mm/hr, were within normal limits, and no further surgical interventions were necessary.

## Discussion

Osteomyelitis continues to be a common problem amongst the pediatric population. Calcaneus is an uncommon site with a rate of 3% to 10% of all cases of pediatric osteomyelitis [4]. The Cierny-Mader treatment approach follows a two-stage process: the first stage focuses on comprehensive drainage, debridement, and the obliteration of dead space, with antibiotic-impregnated acrylic beads used to sterilize the site and temporarily maintain the space. After four to six weeks, a second surgery is performed to remove the beads and replace them with a cancellous bone graft to restore bone integrity [5].

The choice of antibiotics depends on the wound flora and its sensitivity to the antibiotic incorporated into the cement. Aminoglycosides and vancomycin are commonly selected for local delivery due to their broad-spectrum activity and resistance to the heat generated during the exothermic polymerization of the cement. In this case, 2 grams of vancomycin were combined with 40 grams of polymethyl methacrylate (PMMA), which is the gold standard for local delivery of the therapeutic agents. This formulation typically ensures an effective release of vancomycin while maintaining adequate mechanical strength [3]. Additionally, this technique enables the delivery of high local concentrations of antibiotics while avoiding the risk of systemic toxicity associated with prolonged intravenous antibiotic use, which is a growing concern due to the emergence of antibiotic resistance [6].

In vitro studies have shown that PMMA cement spacers loaded with aminoglycosides or glycopeptides provide effective antibacterial activity for only four to six weeks [3]. As antibiotic elution decreases over time, the residual PMMA can serve as a substrate for bacterial colonization. Microorganisms readily adhere to PMMA surfaces, and the formation of biofilms is a critical concern, as it significantly increases the risk of persistent or recurrent infection. For this reason, removal of the spacer during a second-stage surgery is often recommended to prevent reinfection. However, this additional surgical procedure is not without risks, including potential complications related to anaesthesia, impaired wound healing, and increased financial burden on both the patient and the healthcare system.

Alternative treatments that eliminate the need for a second-stage procedure include bioabsorbable antibiotic delivery systems. For example, collagen fleece offers effective triphasic antibiotic release, while polyesters degrade more slowly and may provide some intracellular activity. Additionally, calcium-based carriers such as plaster of Paris, calcium sulfate, and calcium hydroxyapatite allow tissue and bone ingrowth as they break down. However, these biodegradable materials have several drawbacks. They lack sufficient strength to serve as load-bearing spacers, and their chemical properties can sometimes prevent proper hardening, limiting the amount of antibiotics that can be incorporated [7]. Furthermore, as these materials degrade, they can release inflammatory byproducts, potentially leading to wound seromas in as many as 20-28% of cases [7].

Our patient did not undergo the second-stage surgery as her family could not afford the procedure and chose to decline further intervention. This decision was also supported by favourable outcomes from other studies, where attempts to remove antibiotic cement spacers were unsuccessful, yet after three years of follow-up, no complications were reported [8]. Our choice to forgo the second-stage surgery is further supported by extensive experience from joint replacement surgeons and the existing literature on the use of PMMA bone cement [9]. The use of cemented femoral stems, particularly in the aging population, has proven to be a safe and increasingly common practice in both elective and non-elective hip arthroplasty [10]. Xu Shengqiu et al. [11] treated eight osteomyelitis patients (seven tibial, one calcaneal) with debridement and PMMA spacer retention. After a two-year follow-up, no cement-related complications were observed. Additionally, Choi et al. reported that 18 patients with periprosthetic joint infections (11 hips, seven knees) who were treated with retained prosthetic articulating spacers showed no complications related to the cement spacer, with an average follow-up of 43.8 months (range, 13-78 months) [12].

Our case supports the notion that, under certain circumstances, the second stage of the contingency management (C-M) treatment approach to chronic osteomyelitis can be omitted, with no complications arising from the long-term retention of PMMA. As previously noted, the use of absorbable biomaterials presents several limitations. Furthermore, these materials are often expensive and may not be widely available in resource-limited settings.

## Conclusions

The use of antibiotic-impregnated PMMA cement has emerged as an effective strategy in the management of chronic calcaneal osteomyelitis. This technique provides prolonged local antimicrobial activity, which helps control infection, while also offering mechanical stability that supports weight-bearing and promotes normal gait and mobility. In select cases-particularly in stable patients who remain asymptomatic and infection-free over a sustained follow-up period, second-stage surgery to remove the cement spacer may be safely omitted. However, this approach should not be considered universally applicable, and careful patient selection and close clinical monitoring are essential.

While these findings support a more conservative, individualized treatment pathway that can reduce surgical burden-especially in low-resource settings-further research is needed to validate long-term outcomes. In particular, there is a pressing need for more pediatric-specific data, extended follow-up studies, and evidence from diverse healthcare environments to guide optimal management strategies.
